# The Mammalian Family of Katanin Microtubule-Severing Enzymes

**DOI:** 10.3389/fcell.2021.692040

**Published:** 2021-08-03

**Authors:** Nicole A. Lynn, Emily Martinez, Hieu Nguyen, Jorge Z. Torres

**Affiliations:** ^1^Department of Chemistry and Biochemistry, University of California, Los Angeles, Los Angeles, CA, United States; ^2^Molecular Biology Institute, University of California, Los Angeles, Los Angeles, CA, United States; ^3^Jonsson Comprehensive Cancer Center, University of California, Los Angeles, Los Angeles, CA, United States

**Keywords:** katanin, microtubule-severing, microtubule-based structures, cell division, cilia

## Abstract

The katanin family of microtubule-severing enzymes is critical for cytoskeletal rearrangements that affect key cellular processes like division, migration, signaling, and homeostasis. In humans, aberrant expression, or dysfunction of the katanins, is linked to developmental, proliferative, and neurodegenerative disorders. Here, we review current knowledge on the mammalian family of katanins, including an overview of evolutionary conservation, functional domain organization, and the mechanisms that regulate katanin activity. We assess the function of katanins in dividing and non-dividing cells and how their dysregulation promotes impaired ciliary signaling and defects in developmental programs (corticogenesis, gametogenesis, and neurodevelopment) and contributes to neurodegeneration and cancer. We conclude with perspectives on future katanin research that will advance our understanding of this exciting and dynamic class of disease-associated enzymes.

## Introduction

Discovered in sea urchin eggs in 1993 and named after the Japanese expression for sword (katana), the katanins are a family of microtubule-severing enzymes (McNally and Vale, 1993) belonging to the ATPases Associated with diverse cellular Activities (AAA+) protein superfamily (Snider et al., 2008). Katanins function by harnessing the energy produced from ATP hydrolysis to drive microtubule-severing events (McNally and Vale, 1993; McNally and Thomas, 1998). Due to their role in facilitating cytoskeletal rearrangements, the katanins have become the subject of intense research in the fields of neuroscience (Banks et al., 2018), cancer (Wang et al., 2020), and cell and structural biology (Willsey et al., 2018; Faltova et al., 2019; Korulu and Yildiz, 2020).

Katanin is a heterodimeric complex composed of the catalytic ATPase containing A-subunit (p60, KATNA1) and regulatory B-subunit (p80, KATNB1) (McNally and Vale, 1993; Hartman et al., 1998). While the p60 (A) and p80 (B) katanin subunits are conserved in eukaryotes with regard to protein sequence and function (McNally and Thomas, 1998; Srayko et al., 2000), the genomes of vertebrates encode multiple A- and B-subunits. As a testament to the importance of katanins in cellular homeostasis, dysregulation of katanin A- or B-subunit function is associated with developmental and proliferative disorders in vertebrates (Mishra-Gorur et al., 2015; Willsey et al., 2018) and mammals (Bartholdi et al., 2014; Stessman et al., 2017; Banks et al., 2018). For example, perturbation or loss of katanin subunit expression contributes to ciliopathies (Hu et al., 2014; Willsey et al., 2018), defective corticogenesis (Lombino et al., 2019), defective spermiogenesis (Smith et al., 2012; Pleuger et al., 2016), and cancer pathogenesis (Fu et al., 2018; Ye et al., 2020).

In this review, we focus on the mammalian family of katanin proteins: how they function, how they are regulated, and how their dysregulation can lead to an array of human diseases. We examine the function of katanin in shaping diverse microtubule-based structures during interphase, cell division, and ciliation in post-mitotic (terminally differentiated) cells and in post-meiotic cells during spermiogenesis. Furthermore, we contextualize the importance of katanins’ diverse functions with its dysregulation in altered cellular states, disease pathologies, and developmental disorders. Similarly, the structural studies that have informed on the catalytic activity and function of katanins and the mechanisms by which katanins are regulated (genetic, transcriptional, and posttranslational) will be discussed. We conclude with future directions and perspectives on this exciting class of disease-associated microtubule-severing enzymes.

## Katanin Subunit Conservation and Domain Organization

**FIGURE 1:**
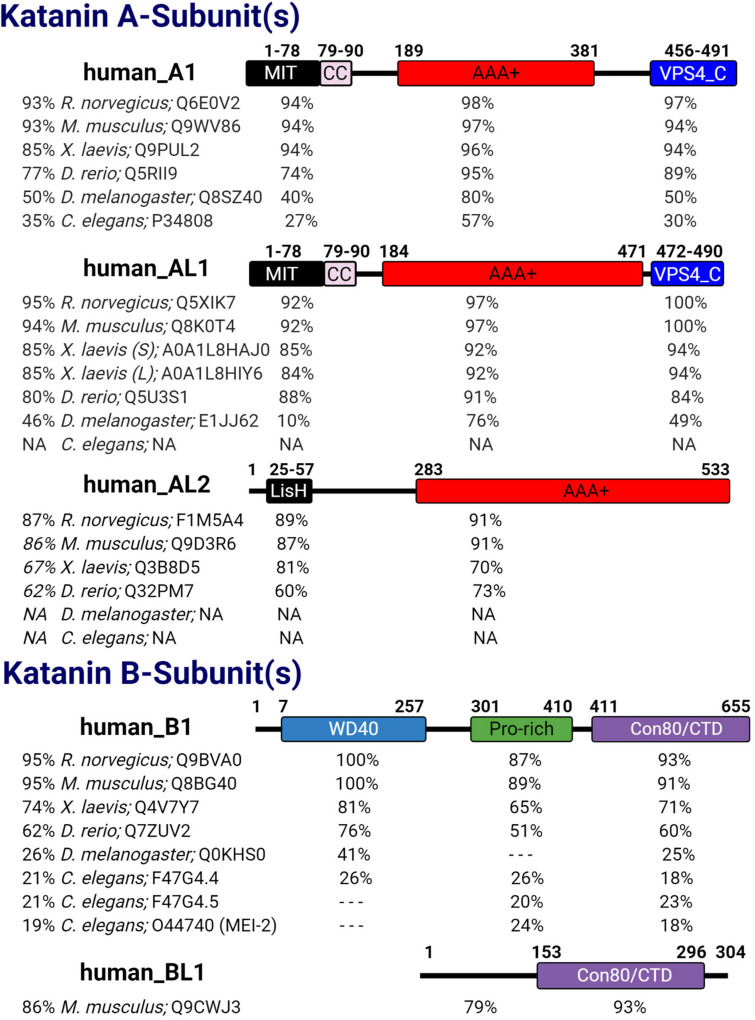
Katanin subunit conservation. The five human katanin subunits—A1 (UniProt ID: O75449), AL1 (UniProt ID: Q9BW62), AL2 (UniProt ID: Q8IYT4), B1 (UniProt ID: Q9BVA0), and BL1 [UniProt ID: Q9H079 (human)]—and their corresponding domains are displayed. For each human katanin subunit, the relative percent identity to full-length (FL) and to each domain therein is indicated for its orthologs in other organisms. Percent identity was determined using NCBI protein blast with BLOSUM62 matrix. Dashed lines indicate no percent identity for that region; N/A indicates that the species lacks that homolog. For *Xenopus laevis* AL1, S and L indicate short and long isoforms, respectively. For *Caenorhabditis elegans* B1 homologs F47G4.4 and F47G4.5 are WormBase IDs and a UniProt ID was used for MEI-2.

Katanin is a heterodimeric protein composed of a catalytic p60 A-subunit and a regulatory p80 B-subunit. While the A-subunit is capable of severing microtubules alone (Hartman and Vale, 1999; Dunleavy et al., 2017; Rezabkova et al., 2017), the binding of the B-subunit to the A-subunit regulates the intracellular localization and activity of the A-subunit (Hartman et al., 1998; Cheung et al., 2016). The mammalian katanin A-subunit KATNA1 (A1) is evolutionarily conserved in unicellular eukaryotes, invertebrates such as *Caenorhabditis elegans* and *Drosophila melanogaster*, and vertebrates including *Danio rerio*, *Xenopus laevis*, *Mus musculus*, *Rattus norvegicus*, and *Homo sapiens* (Figure 1). Additional katanin A-like subunits KATNAL1 (AL1) and KATNAL2 (AL2) are also present in unicellular eukaryotes, *Drosophila*, and vertebrates (Figure 1). While the katanin B-subunit KATNB1 (B1) is conserved from *C. elegans* to *H. sapiens*, the katanin B-like subunit KATNBL1 (BL1) is not as widely conserved (Cheung et al., 2016; Figure 1). Interestingly, nematodes also contain two variants of the canonical B-subunit where the N-terminal WD40 domain is removed (McNally and Roll-Mecak, 2018; Figure 1). The presence of additional A-like katanin subunits among eukaryotes and the additional B-like subunits observed in vertebrates alludes to an increased complexity in microtubule severing that is required for cellular homeostasis.

Katanin subunits share several important domains that are critical to their heterodimerization (A–B dimers), homo-oligomerization (A–A oligomers), binding to microtubules and other proteins, and microtubule-severing function (Figure 1). For example, katanin A-subunits share a catalytic AAA+ domain that contains the conserved Walker A and B motifs (hereafter referred to as pore loops 1 and 2), which coordinate ATP binding and hydrolysis, respectively (Snider et al., 2008; Zehr et al., 2017; Nithianantham et al., 2018). Structural models indicate that upon A-subunit hexamerization, the pore loop 1 X-R-G motif creates a positively charged surface at the entrance to the catalytic site that functions to recognize and remodel tubulin (Zehr et al., 2017; Shin et al., 2019).

Katanins first discovered functions during meiosis placed them into the meiotic clade of AAA+ proteins, which includes microtubule-severing enzymes such as fidgetin and spastin, as well as VPS4, an ESCRT disassembly and membrane remodeling protein (Monroe and Hill, 2016). Outside of the AAA+ domain, KATNA1 and KATNAL1 are nearly identical, sharing the N-terminal microtubule interacting and trafficking (MIT) and C-terminal VPS4_C domains, which are both absent in KATNAL2. The importance of the VPS4_C domain to KATNA1 and KATNAL1 in microtubule severing has yet to be assessed; however, eukaryotic proteins with VPS4_C domains exhibit roles in mediating membrane remodeling/fission and protein degradation (McCullough et al., 2018). In addition to binding to the microtubule lattice, the MIT domain of KATNA1 and KATNAL1 mediates their binding to KATNB1 (Hartman and Vale, 1999; McNally et al., 2000; Cheung et al., 2016). Interestingly in KATNAL2, the MIT domain of KATNA1 and KATNAL1 is replaced by a lissencephaly homology (LisH) domain (Figure 1; Cheung et al., 2016; Ververis et al., 2016). In the ciliate *Tetrahymena thermophila*, the LisH domain of the katanin homolog Kat2 is important for its stability, self-dimerization, and localization to the basal body and ciliary axoneme (Joachimiak et al., 2020). Although the functional importance of the KATNAL2 LisH domain has yet to be determined in vertebrates, it is likely to regulate KATNAL2 localization, stabilization, self-association, and association with other proteins.

The archetypal regulatory katanin B-subunit contains an N-terminal WD40 domain followed by a proline-rich region and a conserved C-terminal domain (denoted con80 or KATNB1-CTD) (Figure 1). The WD40 domain directs A-subunit localization to the spindle poles of the cell (McNally et al., 2000). The proline-rich region of KATNB1 has yet to be assessed; however, eukaryotic proteins with proline-rich regions have roles in transcription, cytoskeletal rearrangements, and intracellular signaling (Kay et al., 2000). The con80 domain of KATNB1 is required for binding to the A-subunit and regulation of its microtubule-severing activity (Hartman et al., 1998; McNally et al., 2000). Recent co-immunoprecipitation studies in HEK293 cells and in elongating spermatids revealed that KATNAL2 was capable of interacting with KATNB1 (Dunleavy et al., 2017). This interaction is intriguing as previous studies in HeLa cells failed to detect an interaction between KATNAL2 and KATNB1 (Cheung et al., 2016), suggesting that this interaction may be cell type specific. How this KATNAL2–KATNB1 interaction is mediated is still unknown, as KATNAL2 lacks the canonical MIT domain. It is possible that this association is mediated through the KATNAL2 LisH domain in the same manner that the KATNA1 MIT and KATNB1 con80 domains interact, as LisH replaces the MIT (McNally et al., 2000). Alternatively, the KATNAL2 LisH domain may interact with the WD40 domain of KATNB1, as WD40 domains can mediate protein–protein interactions (Xu and Min, 2011). In support of this idea, previous studies with LisH and WD40 domain containing proteins showed that the LisH–WD40 interaction promoted oligomerization (Choi et al., 2008). In this regard, the KATNAL2 LisH domain may function to facilitate higher-order oligomerization with the katanin B-subunit. Therefore, defining the nature of the KATNAL2–KATNB1 interaction will inform on the ability of KATNB1 to modulate KATNAL2 activity and/or affect its localization. Interestingly, KATNBL1 lacks the N-terminal WD40 and proline-rich domains found in KATNB1 and only maintains the C-terminal con80 domain, which is necessary and sufficient for binding to and regulating KATNA1 and KATNAL1 microtubule-severing activity (Figure 1; Cheung et al., 2016).

## Katanin Higher-Order Structures

Homohexameric KATNA1 was first observed in the sea urchin *Strongylocentrotus purpuratus* by electron microscopy (EM), where it displayed ring-like structures 14–16 nm in diameter (Hartman et al., 1998). Hexamerization occurs via the AAA+ domain, and further characterization of KATNA1 led to the discovery that oligomerization stimulates catalytic activity and increases microtubule affinity (Hartman and Vale, 1999). Subsequent studies using X-ray diffraction, solution small-angle X-ray scattering, and cryo-EM structures of full-length *C. elegans*, human KATNA1, and human KATNAL1 have confirmed their homohexameric assembly (Zehr et al., 2017, 2020; Nithianantham et al., 2018). Stable KATNA1 oligomerization occurs in an ATP- and microtubule-dependent manner. Here, the microtubule acts as a scaffold that promotes interactions necessary for higher-order assembly, while binding to ATP is proposed to enhance hexamer stability (Hartman and Vale, 1999). Recent studies have shown that oligomerization is also dependent upon KATNA1 concentration: at low levels (< 24 μM) KATNA1 is largely monomeric; however, at increased concentrations (> 25 μM and higher), hexamerization is observed (Zehr et al., 2017; Shin et al., 2019). Although these structural studies have advanced our understanding of the structure and function of KATNA1 and KATNAL1, especially the critical and conserved AAA+ domain, there are currently no structures available for KATNAL2. The KATNAL2 AAA+ domain shares about 50% identity with that of KATNA1 and KATNAL1; therefore, determining its structure will inform on the functional conservation or divergence of this poorly characterized A-like subunit.

In addition to the available homohexameric structures, various structures of the heterodimeric katanin A–B complex have been solved and have defined the KATNA1:MIT and KATNB1:CTD (aka con80) interaction. For example, the mouse KATNA1-MIT:KATNB1-CTD structure (PDB ID: 5NBT) showed the formation of a tight heterodimeric complex relying on interactions between residues R516 and Y519 of the KATNB1-CTD with S75 and K77 of the KATNA1-MIT, respectively (Rezabkova et al., 2017). When overexpressed in cells, the KATNA1-MIT:KATNB1-CTD heterodimeric complex decorates microtubule ends, causing bending and breakage; disrupting the formation of this complex prevents KATNB1-CTD from recruiting KATNA1-MIT to microtubules (Rezabkova et al., 2017). Another mouse-derived KATNA1-MIT:KATNB1-CTD structure (PDB: 6GZC) revealed the formation of a heterotetramer (dimer of heterodimers) (Faltova et al., 2019). Here, heterotetramer formation was shown to limit the accessibility of key residues required for microtubule end-binding compared with the heterodimeric complex. As a heterotetramer KATNA1-MIT:KATNB1-CTD lacks the ability to bind to microtubule plus ends; however, when tested on stable microtubules, its lattice binding and microtubule-severing activities were enhanced upwards of 17-fold compared with heterodimeric katanin (Faltova et al., 2019). These studies suggest that katanin can exist in multiple conformations within the cell and that these conformations are reflective of katanin’s multiple functions. Lastly, despite the increased progress on solving various heterodimeric structures of the KATNA1-MIT:KATNB1-CTD, a structure of the katanin holoenzyme in complex with the microtubule has not been resolved, nor has any single subunit been observed in complex with a microtubule. These structures could further inform on katanin assembly and function on the lattice and at other microtubule regions, such as the plus and minus ends.

## Katanin Function in Cycling Cells

**FIGURE 2:**
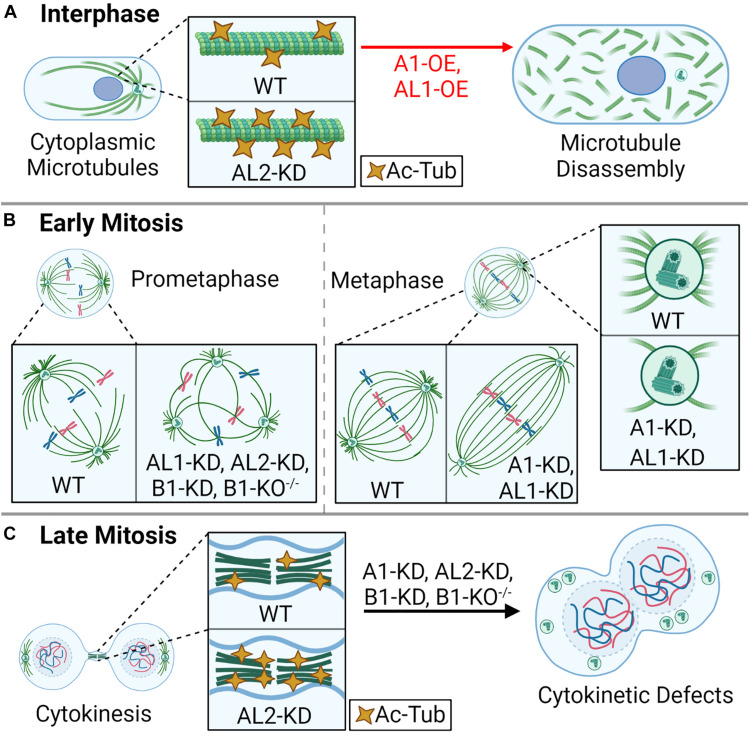
Katanin function in the cell cycle. During the cell division cycle, the katanins are important for cytoplasmic microtubule rearrangements, mitotic spindle assembly, and proper cytokinesis. **(A)** In interphase, overexpression (OE) of A1 or AL1 leads to rapid disassembly of the microtubule lattice, and AL2 knockdown (KD) leads to hyper-acetylated tubulin (Ac-Tub). **(B)** In early mitosis (prometaphase), KD of AL1, AL2, or B1, or homozygous knockout of B1 (B1-KO^– /–^) leads to multipolar spindles; KD of A1 or AL1 leads to an elongated spindle; KD of A1 or AL1 also leads to a reduction of aster microtubule density at the spindle poles. **(C)** In late mitosis, KD of A1, AL2, or B1, or homozygous knockout of B1 (B1-KO^– /–^) leads to cytokinesis failure, enlarged cells with large nuclei, and supernumerary centrioles. KD of AL2 also leads to hyper-Ac-Tub at intercellular bridge microtubules.

The mammalian cell cycle is composed of interphase (G_1_, S, and G_2_ phases), where a cell grows and duplicates its DNA, and mitosis (M phase), where a cell equally segregates its DNA into two daughter cells (Wenzel and Singh, 2018). During interphase, cytoplasmic microtubules extend radially from the centrosome, an organelle that acts as the microtubule organizing center, which is composed of two microtubule-based centrioles surrounded by a matrix of proteins (pericentriolar matrix) (Cooper, 2000; Goodson and Jonasson, 2018). The microtubule cytoskeleton network is essential for shaping the cell, intracellular trafficking, and cell motility (Fletcher and Mullins, 2010). The katanins are critical for regulating the length and dynamics of cytoskeletal microtubules through their microtubule-severing activity, and katanin dysregulation can lead to errors in cell shape, cell migration, cell cycle progression, and cell proliferation (Buster et al., 2002; O’Donnell et al., 2012; Smith et al., 2012; Lombino et al., 2019). KATNA1, KATNAL1, KATNAL2, and KATNB1 localize diffusely to cytoplasmic microtubules and concentrate at the centrosomes (Hartman et al., 1998; McNally and Thomas, 1998; Cheung et al., 2016; Ververis et al., 2016; Willsey et al., 2018). In contrast, KATNBL1 localizes to the nucleus in interphase via an N-terminal nuclear localization signal (Cheung et al., 2016), which suggests that KATNBL1 may have important roles independent of its microtubule-severing activity and/or that its sequestration in the nucleus is a regulatory mechanism to keep cytoplasmic microtubule severing in balance. In support of this idea, overexpression of KATNA1 or KATNAL1 increases cytoplasmic microtubule severing (Figure 2A; McNally et al., 2000; Sonbuchner et al., 2010), and KATNBL1 has been shown to modulate KATNA1 and KATNAL1 activity *in vitro* (Cheung et al., 2016), similar to the observed regulation by KATNB1 (McNally et al., 2000). However, KATNBL1-based regulation is concentration dependent: enhancing activity at low concentrations while inhibiting activity at increasing or equimolar concentrations (Cheung et al., 2016).

In addition to their effect on cytoplasmic microtubules, modulating the expression of the katanin A-subunits also influences cell cycle progression. For example, cells depleted of KATNA1 or KATNAL2 accumulate in G2/M phase with enlarged cytoplasmic volumes (Matsuo et al., 2013; Ververis et al., 2016). KATNAL2 depletion also leads to an increase in acetylated microtubules, which is a hallmark of microtubule stability (Figure 2A; Matsuo et al., 2013; Ververis et al., 2016). The G2/M checkpoint is critical for ensuring that DNA and cellular damage is repaired prior to mitotic entry (Chao et al., 2017). Furthermore, the G2–M transition marks the end of cell growth and the beginning phase of mitosis (prophase). This transition is marked by an acute decrease in microtubule polymer and an increase in microtubule dynamics to initiate the necessary structural changes, for example, the breakdown of the nuclear envelope (Zhai et al., 1996). Therefore, it is possible that the perturbation of microtubule dynamics through a decrease in microtubule severing and/or an increase microtubule stabilization via acetylation, seen upon depletion of KATNA1 or KATNAL2, are sensed as damage by the G2/M checkpoint.

The active process of cell division involves mitosis, where a cell’s DNA is equally distributed to two nascent daughter cells, and cytokinesis, where the cytoplasm is bisected to generate two distinct daughter cells (Poon, 2016). During mitosis, katanins are primarily responsible for regulating the size and shape of the mitotic spindle through their functions at the centrosome (McNally and Thomas, 1998) and spindle (Loughlin et al., 2011). At the centrosome, KATNA1 recruits γ-tubulin, which is required for nucleating microtubules and generating microtubule density; inhibition of KATNA1 significantly reduces spindle density in prometaphase, concomitant with the loss of γ-tubulin (Figure 2B; Buster et al., 2002). In *X. laevis* and *Xenopus tropicalis*, KATNA1 regulates the length of mitotic and meiotic spindles as well as the length of k-fibers, which attach to chromosomes (Loughlin et al., 2011). Depletion of KATNA1 or KATNAL1 leads to similar spindle defects, including reduced spindle pole density and an increase in spindle length (Figure 2B; Sonbuchner et al., 2010). In contrast to KATNA1 and KATNAL1, KATNAL2 is more abundant along the mitotic spindle (Cheung et al., 2016; Willsey et al., 2018). Despite slight differences in localization, knockdown of KATNAL2 in mammalian cells phenocopies that of KATNAL1 and KATNB1, leading to the increased production of multipolar spindles (Figure 2B; Hu et al., 2014; Ververis et al., 2016; Gao et al., 2019). The different domain composition of KATNAL2 compared with KATNA1 and KATNAL1 suggests that KATNAL2 localization and effect on the mitotic spindle may be driven by other factors, which could include LisH-mediated protein–protein interactions.

As mitosis progresses, the katanins are dynamically redistributed from the spindle and spindle poles to other microtubule-based structures. For example, during telophase, KATNA1 localizes to the gap between the contractile ring and central spindle bundle, and at microtubules flanking the midbody; this distribution is independent of the B-subunit (Matsuo et al., 2013). Furthermore, KATNA1 knockdown leads to cytokinesis failure and an increase in binucleate cells (Figure 2C; Matsuo et al., 2013). While proteins like ASPM can complex with KATNA1 and KATNB1 to regulate microtubule-severing activity at the spindle poles and on the microtubule lattice (Jiang et al., 2017), little is known about the protein interactions that regulate KATNAL1 and KATNAL2. For example, KATNAL2 localizes to the midbody during cytokinesis, and its depletion leads to an increase in microtubule acetylation at the midbody, as well as the production of chromosome bridges, multinucleated cells, and apoptosis (Figure 2C; Ververis et al., 2016; Willsey et al., 2018), but little is known about how this occurs. KATNB1 also has a dynamic cell cycle phase-dependent subcellular localization, localizing to the cytoplasm and nucleus during interphase, the spindle midzone in anaphase, and to sister chromatids in cytokinesis (Suko and Maru, 2007; Jin et al., 2017). The variable and dynamic localization of katanin A- and B-subunits in the later stages of mitosis highlight a potential for subunit-specific functions. Similarly, variable A- and B-subunit localization has been observed in ciliates such as *Tetrahymena*, which may be explained by differences in A–B-subunit expression (Waclawek et al., 2017). It is also possible that changes in localization are driven by KATNB1-specific interactions; for example, in mammals, KATNB1 binds to Lis1, a protein known to localize to the kinetochore microtubules, which does not interact with KATNA1 (Toyo-Oka et al., 2005). Alternatively, the differences in localization of the katanin A- and B-subunits could be due to improperly validated reagents, such as the antibodies used.

Outside of their mitotic functions, katanins are also critical for meiotic cell divisions. During *C. elegans* meiosis I, the katanin A-subunit (MEI-1) and B-subunit (MEI-2) are critical for spindle organization, assembly, and shortening (Srayko et al., 2000, 2006; McNally et al., 2006). Interestingly, unlike the A-subunits of sea urchins and vertebrates, which can sever microtubules independent of the B-subunit, the activity of *C. elegans* MEI-1 is contingent upon MEI-2 (Hartman et al., 1998; McNally et al., 2006; Joly et al., 2016). During meiosis II, the MEI-1/MEI-2 complex is essential for central spindle disassembly, where microtubules between polar bodies and the female pronucleus are severed (McNally et al., 2006; Gomes et al., 2013). Prior to fertilization, *C. elegans* oocytes arrest in meiosis; fertilization triggers the rapid completion of meiosis and the transition to mitotic divisions (DeRenzo and Seydoux, 2004). During this oocyte-to-embryo transition, elimination of MEI-1 and MEI-2 is required to progress from the last meiotic division to the first mitotic division; failure to do so results in spindle defects and embryonic lethality (Srayko et al., 2000; Lu and Mains, 2007). In mouse spermatocytes, mutations that render KATNB1 dysfunctional promote stalls in anaphase I and an increase in the frequency of binucleate cells (O’Donnell et al., 2012). In mouse oocytes, KATNAL1 is essential for ensuring spindle pole integrity during meiosis I and II; modulating KATNAL1 influences oocyte maturation and fertility (Gao et al., 2019). However, the roles (if any) of the other mammalian A/B katanin subunits in meiosis remain to be determined. The varied spatial and functional requirement of specific katanin subunits for regulating diverse microtubule-based structures in specialized settings highlights their capabilities for sub-specializations during mitotic and meiotic cell divisions.

## Katanin in Ciliary Homeostasis and Development

**FIGURE 3:**
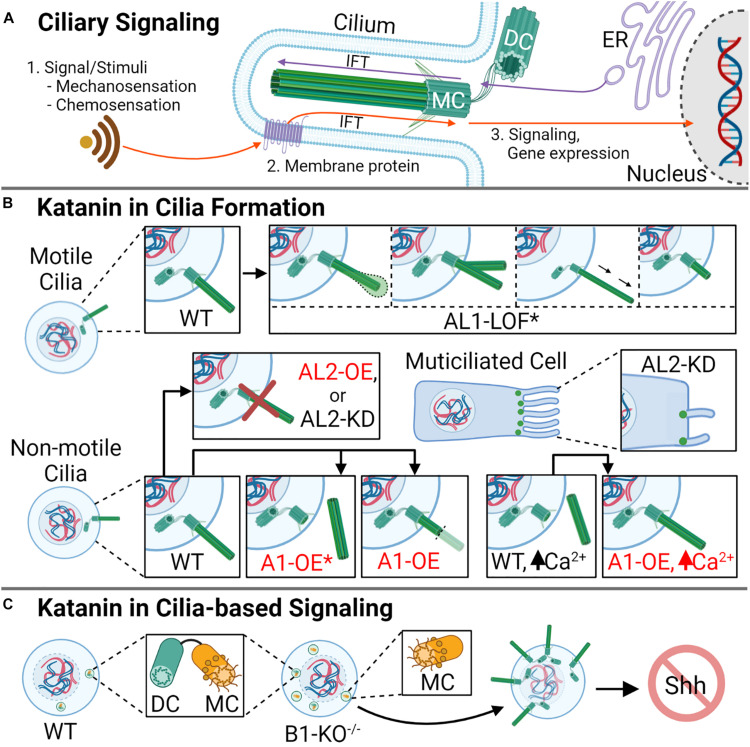
Katanin function in ciliogenesis and cilia resorption. **(A)** Cilia are important microtubule-based organelles important for cell signaling during development. **(B)** In motile cilia, AL1-LOF* (asterisk denoting the mutation AL1^1H/1H^) causes a variety of defects, including swelling at the ciliary tip, ciliary bifurcation, elongation, and shortening, causing defective ciliary movement. In non-motile ciliated cells, A1 overexpression (OE) promotes cilia disassembly, with rapid deciliation as the favored method (indicated by asterisk). In cells with high levels of Ca^2+^, which signals deciliation, A1-OE prevents the loss of cilia. Knockdown or overexpression of AL2 in non-motile cilia causes a reduction in ciliogenesis, while in multiciliated cells, AL2 KD reduces cilia number and reduces cilia length. KD of AL2 to levels below 50% or AL2 OE triggers apoptosis (not shown). **(C)** Homozygous knockout of B1 (B1-KO^– /–^) leads to the generation of supernumerary centrioles, excess mother centrioles, aberrant ciliation, and disrupted cellular signaling, including sonic hedgehog (Shh) signaling. DC indicates daughter centriole, and MC indicates mother centriole.

Cilia are microtubule-based organelles that protrude from most mammalian cells and are either non-motile (primary) or motile (Ishikawa and Marshall, 2011; Malicki and Johnson, 2017). The assembly of primary cilia initiates in G_1_ phase, and growth continues as cells exit into G_0_ (Malicki and Johnson, 2017). G_0_ encompasses a phase of the cell cycle where cells are not actively dividing (quiescent) but have the potential to re-enter division; this phase is also used to describe cells that have become terminally differentiated (TD), such as neurons (Schafer, 1998). During cell cycle re-entry, primary cilia resorption occurs before the G_1_/S transition, although some cells exhibit cilia of minimal length during S phase (Plotnikova et al., 2009). Primary cilia are essential for signaling processes like chemosensation, osmosensation, and phototransduction (Waters and Beales, 2011; Figure 3A). Motile cilia, on the other hand, are important during mammalian development to establish unidirectional flow of extraembryonic fluid, which is subsequently required for left–right patterning (Hirokawa et al., 2009). Furthermore, specific populations of cells in the lungs, oviducts, and brain ventricles are multiciliated and can form up to 300 motile cilia (Meunier and Azimzadeh, 2016). Due to the importance of cilia during development and in organismal homeostasis, ciliary dysfunction is associated with a broad array of diseases that affect development, reproduction, and organ function, which are collectively known as ciliopathies (Waters and Beales, 2011).

The function of katanin on the cilia has been widely studied using unicellular eukaryotes; for example, research investigating *Tetrahymena* and *Chlamydomonas* A- and B-subunit homologs has informed on katanin activity and localization on ciliary structures (Waclawek et al., 2017; Joachimiak et al., 2020), as well as the biogenesis of motile cilia (Sharma et al., 2007). Several recent studies have implicated katanin in the assembly, disassembly, and function of cilia in vertebrates (Hu et al., 2014; Willsey et al., 2018; Mirvis et al., 2019). For example, in mouse kidney cells, KATNA1 localizes to the base of the primary cilium, and its overexpression induces both rapid and gradual loss of cilia, but rapid deciliation is favored (Figure 3B; Mirvis et al., 2019). These findings are intriguing as *Tetrahymena* does not require the A- or A-like subunits for stress-induced deciliation compared with vertebrates (Sharma et al., 2007), while *Chlamydomonas* requires the katanin A-subunit to induce deciliation, specifically severing the axoneme (Lohret et al., 1998), suggesting that the katanins in vertebrate deciliation may be more similar to that of *Chlamydomonas*. In comparison with KATNA1, KATNAL2 localizes to the ciliary axoneme, basal body, and daughter centriole; furthermore, its knockdown leads to a 50% reduction in ciliated cells in mice (Ververis et al., 2016). In the multiciliated embryonic epithelial cells of *X. tropicalis*, KATNAL2 is required for ciliogenesis, and knockdown results in ciliary shortening and a reduction of cilia number (Figure 3B; Willsey et al., 2018). In motile cilia, such as those observed in mouse ependymal cells, KATNAL1 is important in cilia maintenance and function. For example, a KATNAL1 loss-of-function (LOF) mutation within the AAA+ domain (L286V, designated KATNAL1^1H/1H^) leads to cilia bifurcation, bending, increases and decreases in ciliary length, and swollen ciliary tips (Figure 3B), abnormalities that affect beat frequency (Banks et al., 2018). These studies indicate that the A-subunits are critical players in ciliogenesis and cilia maintenance and function. However, the role of the B-subunits in these ciliary processes remains to be explored further among vertebrates; such exploration should inform on what katanin A–B complexes are the most important within a particular ciliary context.

The Hedgehog (Hh) signaling pathway is critical for embryo axial body patterning, limb patterning, and organogenesis (Corbit et al., 2005; Komiya and Habas, 2008; Jia et al., 2015; Bangs and Anderson, 2017). Hh signaling is reliant on functional cilia to signal key cellular events like differentiation, growth, and tissue patterning (Huangfu and Anderson, 2005; Nikonova and Golemis, 2015). For example, Hh proteins interact with membrane receptors on the primary cilia such as Patched1 (PTCH1) or Patched 2 (PTCH2), which trigger an accumulation of downstream activating proteins like Smoothened (SMO) that recruits GLI family zinc finger 1 (GLI1) (Raleigh and Reiter, 2019) and influence gene expression programs (Kalderon, 2002). Due to the importance of katanin in ciliogenesis and ciliary maintenance, katanin A- and B-subunits have been implicated in a variety of cilia-dependent signaling pathways including Hh (Hu et al., 2014), Wnt (Willsey et al., 2018), and left–right signaling (Furtado et al., 2017). For example, the complete loss of KATNB1 (KATNB1^–/–^) in mouse embryonic fibroblasts (MEFs) leads to centriole overduplication, an increased presence of mother centrioles, aberrant ciliogenesis, and defective Hh signaling (Hu et al., 2014; Figure 3C). These ciliary perturbations result in reduced expression of downstream sonic hedgehog (Shh) pathway targets GLI1 and Patched; KATNB1^–/–^ mice undergo lethality by embryonic day 15.5 (E15.5), which is the final week of prenatal growth where cardiovascular, palate, and musculoskeletal development occur (Hu et al., 2014; Mishra-Gorur et al., 2015). This outcome is phenotypically similar to that of mice lacking *C2CD3*, a gene encoding a regulatory Hh protein required for GLI3 processing during embryonic development (Hoover et al., 2008). Although it is apparent that katanin’s role in ciliary maintenance affects signaling, it remains unknown whether katanin loss/defects influences intracellular crosstalk between signaling pathways, for example, crosstalk between Wnt and Hh. Similar to their roles in Hh developmental pathways, the katanins are also important for the Wnt/Planar cell polarity (PCP) pathway. For example, KATNAL2 is enriched in multiciliated cells of the developing brain and organs in *X. laevis*; here, LOF or depletion of KATNAL2 disrupts ciliogenesis and cilia maintenance and promotes defects in neural crest migration, blastopore closure, and the disorganization of apical actin (Willsey et al., 2018). Further studies are required to determine if the associated outcomes are due to KATNAL2 dysfunction in ciliary homeostasis/maintenance, the effect of KATNAL2 loss on the Wnt/PCP pathway independent of its role on the cilium, or both. KATNB1 is also ubiquitously expressed throughout embryonic development, with a strong presence in the node pit and crown cells of the developing embryo (Furtado et al., 2017). Upon homozygous loss (KATNB1^–/–^), mice display left–right cardiac malformations (atrial and ventricular septal defects, heterotaxy, and axial distortions) during embryonic development (Furtado et al., 2017). The KATNB1-specific cardiac anomalies were phenotypically similar to those observed upon the loss of *Pixt2*, which is a critical transcription factor in left–right and Wnt signaling pathways (Chinchilla et al., 2011). Intriguingly, the Wnt pathway has been demonstrated to regulate LR patterning in the node of mouse embryos (Kitajima et al., 2013), which suggests that the observed KATNB1 cardiac abnormalities may be more related to the Wnt pathway. Whether KATNB1, KATNBL1, or the katanin A-subunits have direct roles in regulating Wnt signaling and LR patterning, or if their effect on these pathways are a result of their function in ciliogenesis, remains to be determined.

## Katanin Dysfunction in Corticogenesis

**FIGURE 4:**
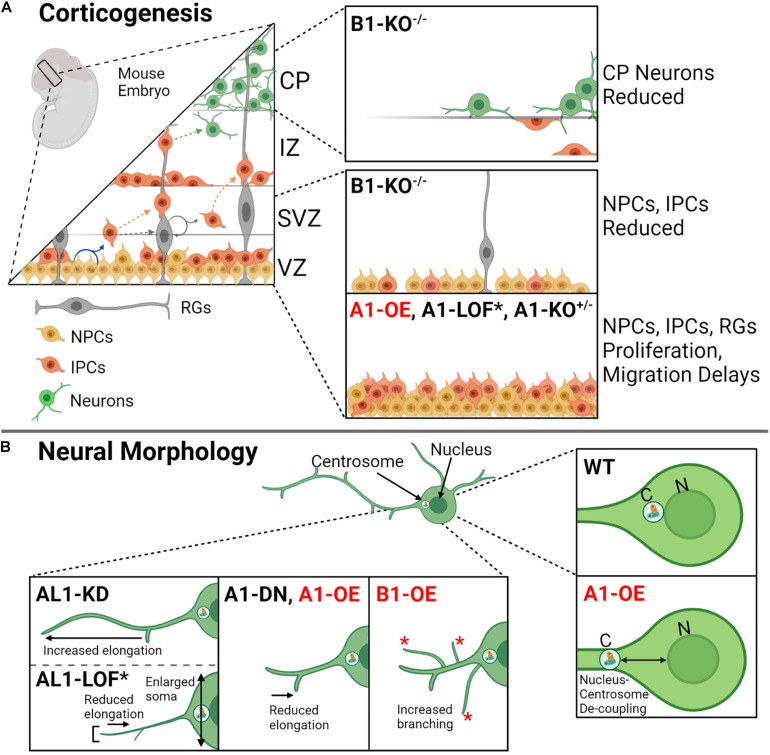
Katanin function during corticogenesis and in neural morphology. **(A)** During mouse corticogenesis, the katanins regulate asymmetrical proliferation, cellular migration, and neuron development. Dysregulation of katanin subunit levels by heterozygous knockout (KO^+/–^), homozygous knockout (KO^– /–^), loss of function (LOF), and/or overexpression (OE) leads to disruptions at different cortical layers (VZ, ventricular zone; SVZ, subventricular zone; IZ, intermediate zone; CP, cortical plate; MZ, marginal zone). The affected cells include neural progenitor cells (NPCs), intermediate progenitor cells (IPCs), radial glia, and developed neurons. **(B)** Katanin A1, AL1, and B1 are involved in neural morphology, contributing to process elongation and branching. Knockdown of AL1 enhances process length (process elongation), while AL1 loss of function by means of a recessive mutation AL1^1H/1H^ in mice (indicated by asterisk; see Figure 3 for this mutant) contributes to shorter and thinned axons, reduced process elongation, and enlarged soma. Dominant negative A1 leads to reduced process elongation as does A1 overexpression. Additionally, A1 overexpression increases the distance in nucleus–centrosome coupling in neurons. Lastly, B1 overexpression promotes increased branching in neurons.

Mature TD cells are classified as post-mitotic, remain in G_0_, and can no longer divide; a classic example of TD cells is neural cells (Ahmad et al., 1999). During corticogenesis, neurons migrate out of the germinal layers to distinct regions within the developing central nervous system (CNS) and become differentiated; neural migration is critical to CNS development, as it provides a mean for cells to interact spatially and reach their final destinations (Figure 4A; Rahimi-Balaei et al., 2018). Katanin expression correlates with axonal development; here, KATNA1 levels increase during axon growth and drop when the axons reach their target or when growth ceases (Karabay et al., 2004). In neurons, katanins have been implicated in generating microtubule fragments, which are trafficked to the cell’s leading edge and neural branches (Ahmad et al., 1999). This function could explain katanins’ varied contributions to neural development, including proliferation (Lombino et al., 2019), migration (Mishra-Gorur et al., 2015), and process elongation (Hatakeyama and Hayashi, 2018). Perturbation of katanin expression levels disrupts these processes. For example, overexpression of KATNA1 in rat neurons inhibits neural migration (Figure 4A), generates defective nucleus–centrosome coupling (Figure 4B), and reduces axon growth (Figure 4B; Toyo-Oka et al., 2005). Similarly, overexpression of dominant-negative KATNA1 leads to a reduction in process elongation (Toyo-Oka et al., 2005; Figure 4B). In contrast to what is observed with KATNA1 overexpression, KATNAL1 knockdown enhances process elongation in Neuro2a cells (Hatakeyama and Hayashi, 2018). Furthermore, a LOF mutation that renders KATNAL1 inactive in mice (KATNAL1^1H/1H^) leads to an increase in neural body (soma) size and shortened thin axons with few dendritic spines (Figure 4B; Banks et al., 2018). Intriguingly, in mice neuronal cells, KATNAL1 is expressed at higher levels and is more stable compared with KATNA1 (Hatakeyama and Hayashi, 2018). Together, these studies suggest that both KATNA1 and KATNAL1 severing activities are important for corticogenesis and neuronal maintenance.

KATNB1 is also highly expressed in neurons (Mishra-Gorur et al., 2015). KATNB1 depletion studies in MEFs and samples derived from patients who harbor mutations that reduce KATNB1 expression showed impaired neuronal migration, reduced neural progenitors, and a reduction of neurons at the cortical plate (Figure 4A; Hu et al., 2014; Mishra-Gorur et al., 2015; Jin et al., 2017). Intriguingly, KATNB1 overexpression in rats increases the number of branches that extend from the neuron, which is in opposition to what is observed when KATNA1 is overexpressed; KATNB1 overexpression instead leads to similar outcomes observed when KATNAL1 is overexpressed (Figure 4B; Yu et al., 2005). This suggests that KATNB1 may preferentially pair with KATNAL1 in neurons, which remains to be tested.

## Katanin Dysfunction in Neurodevelopment

Neurodevelopmental disorders (NDDs) consist of several conditions that often co-occur to impair an individual’s personal, social, and academic functions; these disorders include autism spectrum disorder (ASD), intellectual disabilities (IDs), attention deficit/hyperactivity disorder (ADHD), and communication disorders (Morris-Rosendahl and Crocq, 2020). In humans, haploinsufficiency of KATNAL1 has been correlated with disorders like ID and microcephaly (O’Roak et al., 2012; Bartholdi et al., 2014). Furthermore, KATNAL1 and KATNAL2 were both recently identified as candidate genes in ID and ASD, respectively (O’Roak et al., 2012; Bartholdi et al., 2014; Stessman et al., 2017). In mice, homozygous loss of KATNA1 (KATNAL1^–/–^) compromises neural migration and ventricle size, resulting in ID, impaired learning, memory, and vocalization (Banks et al., 2018). In mice and zebrafish, mutagenesis of KATNB1 at exons 2 and 6, which leads to full deletion and/or the production of a dysfunctional N-terminal truncated protein, respectively, results in gastrulation and forebrain defects during development (anencephaly, microcephaly, and holoprosencephaly) (Hu et al., 2014). Unlike mice and zebrafish, humans with homozygous KATNB1 mutations do not exhibit embryonic lethality (Bartholdi et al., 2014; Hu et al., 2014), suggesting that in humans, KATNB1 mutations may retain partial function or that KATNBL1 may compensate for dysfunctional KATNB1. Together, these studies indicate that the observed perturbations to embryonic development are reflective of the dominant katanin subunits used in a given species and suggest a reliance for specific katanin subunits in specific cell types and/or during specific stages of development.

Neurodegenerative disorders, like Alzheimer’s Disease (AD), are characterized by changes in personality, impaired/decreased judgment, mood disturbances, and progressive dementia (decline in language, memory, and ability to perform basic functions) (Weller and Budson, 2018). Neuropathological hallmarks of AD include the presence of β-amyloid plaques and toxic neurofibrillary tangles (NFTs); in the neurons of AD patients, NFTs are primarily composed of the hyperphosphorylated protein Tau (Lane et al., 2018). Tau is a microtubule-associated protein responsible for stabilizing microtubules along the lattice and growing ends; phosphorylation reduces Tau’s affinity for microtubules, and hyperphosphorylation is associated with neurodegenerative disease (Barbier et al., 2019). Tau-bound microtubules are protected from severing by katanin even in conditions where katanin is overexpressed, while microtubules that lack Tau demonstrate increased sensitivity to katanin-based severing (Qiang et al., 2006). This increased sensitivity to severing by katanin has been proposed as a basis for microtubule loss in tauopathies (Sudo and Baas, 2010, 2011). After Tau loss, the microtubule network rapidly disintegrates, likely as a result of katanin-mediated severing. Other microtubule-severing enzymes are also important for neural cell homeostasis and function, such as Spastin, whose mutations lead to defects in axonal transport and degeneration, and diseases like hereditary spastic paraplegia (HSP) (Leo et al., 2017). Intriguingly, with respect to the protection of microtubules, Tau offers less protection for spastin microtubule severing when compared with katanin (Yu et al., 2008). One example of Tau-based katanin regulation is with KATNAL1, which is overexpressed in the absence of Tau in both MEFs and human mammary epithelial cells (HMECs) (Sudo, 2018). The overexpression of KATNAL1 in Tau’s absence suggests that Tau may modulate katanin expression while also providing protection to microtubules.

The dysregulation of brainstem nuclei has previously been postulated as a primary mechanism for AD pathogenesis (Iatrou et al., 2017). In nuclei derived from the brainstem of AD patients, KATNB1 expression is reduced (Andrés-Benito et al., 2018). This same study showed interactions between KATNB1 and a protein encoded by *KIAA0556*, a gene implicated in AD pathology (Andrés-Benito et al., 2018). These nuclei often contain hyperphosphorylated Tau, which is less protective against katanin severing due to its diminished capacity to interact with microtubules (Brandt et al., 2005; Andrés-Benito et al., 2018). The combination of Tau hyperphosphorylation in conjunction with reduced levels of KATNB1 may therefore create an environment in the cell where the katanin A-subunits can sever microtubules uncontrollably. The mechanism linking KATNB1 dysregulation to AD disease pathology, however, has yet to be explored. Nonetheless, these findings support the idea that an imbalance between Tau and the katanin subunits could contribute to neurological disease progression.

## Katanin Dysfunction in Gametogenesis

**FIGURE 5:**
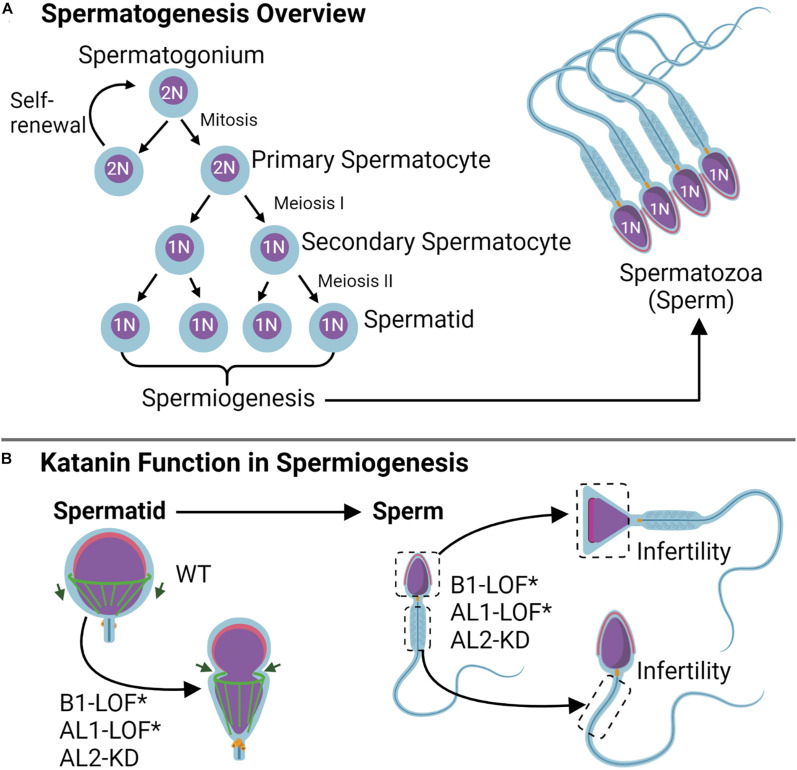
Katanin function in gametogenesis. **(A)** Spermatogenesis overview. During spermatogenesis, the spermatogonium either experiences self-renewal or undergoes two rounds of meiosis to become a spermatid; spermiogenesis occurs after the second meiotic completion and consists of the shaping of the sperm head and tail. **(B)** During spermiogenesis, katanin B1 and AL2 subunits work in shaping the manchette (green) for purposes of head shaping and tail formation. Knockdown (KD) of either AL1 or AL2 as well as B1 loss of function (LOF) by means of the Taily mutation in mice (KATNB1^Taily/Taily^) leads to sperm head and tail shaping defects that cause infertility.

Gametogenesis is the process by which mature sex (germ) cells are formed (Figure 5A). For males, this occurs in two phases and involves the rapid remodeling of complex microtubule structures in meiosis and mitosis to form the spermatogonia and spermatocytes, respectively (Larose et al., 2019). Here, katanin is expressed at high levels and is important for germ cell production (O’Donnell et al., 2012; Pleuger et al., 2016). In Sertoli cells (SCs), testicular nurse cells that aid in spermatogenic development, KATNAL1 regulates microtubule dynamics involved in spermatid adhesion and release, and KATNAL1 LOF leads to male-specific sterility (Figure 5B; Smith et al., 2012; Hatakeyama and Hayashi, 2018). KATNAL2 is similarly involved in SC function (spermatid adhesion/release, acrosome attachment), in addition to directing morphology in spermiogenesis (head shape, tail growth) (Dunleavy et al., 2017). Like the effects of KATNAL1, KATNAL2 knockdown in SCs results in germ cell remodeling defects that lead to male sterility in mice (Dunleavy et al., 2017; Smith et al., 2017; Figure 5B). Of interest, previous studies using mice identified multiple KATNAL2 isoforms that were required for proper ciliogenesis (Ververis et al., 2016) and were expressed during different stages of spermatogenesis and spermiogenesis (Dunleavy et al., 2017). The presence of multiple KATNAL2 isoforms in mice with overlapping ciliary and spermiogenic roles suggests that there may be a need for specific KATNAL2 isoforms during differing stages of these processes.

KATNB1 is also essential for mammalian spermatogenesis (O’Donnell et al., 2012; Pleuger et al., 2016). In mice, KATNB1 functions in shaping the sperm head and developing the flagella during spermiogenesis. A missense mutation in the KATNB1 WD40 domain resulting in the conversion of V to F at position 234 (denoted KATNB1^Taily/Taily^) results in reduced protein levels and a loss of KATNB1 function (O’Donnell et al., 2012). Male KATNB1^Taily/Taily^ mice are infertile, likely due to the production of abnormal meiotic spindles during spermatogenesis (elongated spindles and binucleate spermatids), as well as formation of defective cell structures during spermiogenesis (nuclear distortion, abnormally long microtubules, and axoneme defects in sperm tails) (Figure 5B; O’Donnell et al., 2012). In humans, KATNB1 exhibits variable expression during the different stages of gametogenesis and functions in spermatogonium spindle assembly and in shaping the manchette and flagellum during spermiogenesis (Pleuger et al., 2016). These findings indicate that the katanin holoenzyme is important for establishing male gamete architecture, and this architecture translates to proper function. As such, the katanin holoenzyme has the potential to serve as a novel target for research on male-specific infertility.

## Katanin Dysfunction in Cancer

During carcinogenesis, the signaling pathways required during early development often become aberrantly re-activated, granting cells a proliferative advantage, favored survival, and invasive abilities (Fouad and Aanei, 2017; Nwabo Kamdje et al., 2017). The katanins are expressed during development and throughout life but are aberrantly expressed in multiple types of cancers (Ye et al., 2012; Li et al., 2018). It is well-established that katanin-based microtubule severing is important for proper spindle assembly during cell division and for microtubule rearrangements required for cell migration (McNally and Thomas, 1998; Zhang et al., 2007, 2011). The dysregulation of katanin correlates with errors in these processes and contribute to tumorigenesis and metastasis (Ye et al., 2012, 2020). For example, elevated KATNA1 expression is detectable in metastatic breast and prostate cancers and correlates with enhanced cell migration and reduced proliferation (Table 1; Ye et al., 2012; Fu et al., 2018). Consistently, ectopic KATNA1 overexpression in breast and prostate cancer cells leads to reduced proliferation and enhanced migration, while a reduction in endogenous KATNA1 expression in breast cancer cells enhances proliferation and decreases migration (Table 1; Ye et al., 2012; Fu et al., 2018). The human KATNAL1 mutant L123V identified in breast cancer cells resides in the flexible linker region at the N-terminus of the protein and promotes microtubule-severing activity even in the presence of Tau (Sudo and Nakajima, 2016). The excessive microtubule severing driven by KATNAL1-L123V leads to chromosome bridge formation during cell division and the subsequent formation of micronuclei and aneuploidy, which are associated with breast carcinoma pathogenesis (Sudo and Nakajima, 2016). The elevated expression of KATNB1 shares a similar positive correlation with advanced breast cancer staging, lymph node metastasis, and reduced rates of overall survival in patients (Table 1; Li et al., 2018). Additionally, in non-small cell lung cancer (NSCLC), the elevated expression of KATNA1 and KATNB1 correlates with lymph node metastasis and advanced tumor progression (Table 1; Wang et al., 2020; Ye et al., 2020). Katanin expression is also dysregulated in other types of cancers like papillary thyroid carcinoma (PTC), where both KATNA1 and KATNB1 are highly expressed in tumors and tumor-adjacent tissues; furthermore, elevated katanin expression correlates with advanced PTC staging and worse disease-free survival in patients (Chen et al., 2020). The overexpression of katanin subunits in various cancers and findings that activating mutations in katanin contribute to cancer pathogenesis indicate that katanin dysregulation is an important step in tumorigenesis. As such, advancing our understanding of the connection between katanin holoenzyme dysregulation and cancer pathology could highlight the importance of the katanin enzymes as novel targets for the development of future cancer therapeutics.

**TABLE 1:** Katanin involvement in cancer development.

Katanin	Cancer type	Information	References
KATNA1	Non-small cell lung cancer (NSCLC)	Increased KATNA1 expression correlates with lymph node metastasis and advanced TNM stages.	Wang et al., 2020; PMID: 32631334
	Breast cancer	KATNA1 overexpression promotes cell migration and inhibits cell proliferation; silencing promotes proliferation and inhibits cell migration. KATNA1 expression is significantly increased in primary breast cancer tissue compared with non-cancerous tissue.	Fu et al., 2018; PMID: 29552132
	Prostate cancer	Elevated KATNA1 expression enhances migratory capacity and inhibits cell proliferation. KATNA1 expression in metastatic cells was associated with the re-emergence of basal-cell-like phenotype.	Ye et al., 2012; PMID: 21681775
	Papillary thyroid carcinoma (PTC)	KATNA1 is highly expressed in tumor and tumor-adjacent tissue. Elevated expression correlates with larger tumor size, extrathyroidal invasion, and advanced cancer staging (pT, pN, and TNM stages), as well as worse disease-free survival (DFS) in patients.	Chen et al., 2020; PMID: 33274499
KATNB1	Non-small cell lung cancer (NSCLC)	Elevated KATNB1 expression is associated with larger tumor size, lymph node metastasis, advanced cancer staging (TNM), and decreased rate of disease-free (DFS) and overall patient (OS) survival.	Ye et al., 2020; PMID: 31944409
	Breast cancer	Elevated KATNB1 expression positively correlates with lymph node metastasis and advanced cancer staging (pN and TNM stages) and reduced overall survival (OS).	Li et al., 2018; PMID: 30223388
	Papillary thyroid carcinoma (PTC)	KATNB1 is highly expressed in tumor and tumor-adjacent tissue. Elevated expression correlates with advanced cancer staging (pN stage and TNM stage), and worse disease-free survival (DFS) in patients.	Chen et al., 2020; PMID: 33274499

*TNM is the clinical cancer staging: I, II, III, or IV. T = size, direct extent of tumor; N = degree of spread to lymph; M = presence of distant metastasis; pT or pN = pathological cancer staging.*

TP53 is an important regulator of the cell cycle, proliferation, and apoptosis and is the most widely mutated cancer gene (Fouad and Aanei, 2017). Intriguingly, the TP53 DNA-binding domain (DBD), which functions as a sequence-specific transcription factor (Harms and Chen, 2006), was recently found to bind to the KATNA1 C-terminal region (Korulu and Yildiz, 2020). Furthermore, TP53 is an activator of KATNA1 gene expression. In mammalian HCT116 cells, TP53 was found to bind to the KATNA1 promoter, within the –117 to –95 region, and to upregulate KATNA1 transcription (Kırımtay et al., 2020). Because TP53 is critical for regulating the cell cycle and proliferation, further analysis into the importance of the KATNA1–TP53 interaction will be paramount to understanding tumorigenesis.

## Katanin Regulatory Mechanisms

As is the case with most proteins, katanin abundance, activity, and function is regulated at the transcriptional (Selçuk et al., 2013; Kelle et al., 2019) and posttranslational levels (Cummings et al., 2009; Loughlin et al., 2011; Whitehead et al., 2013). In addition to KATNA1 transcriptional regulation by TP53, KATNA1 and KATNB1 are differentially regulated by the Elk-1 transcription factor. Elk-1 belongs to a family of oncogene transcription factors that activate/repress genes involved in diverse processes like growth, survival, differentiation, proliferation, development, apoptosis, and cancer (Besnard et al., 2011). Elk-1 binds to the 5′ UTR of KATNA1 in a methylation-dependent manner, resulting in reduced KATNA1 expression; this mechanism of regulation is unique to katanin when compared with other severases (Kelle et al., 2019). In contrast, the binding of Elk-1 to the KATNB1 promoter (near but excluding the 5′ UTR) leads to an increase in KATNB1 mRNA and protein levels (Selçuk et al., 2013). The transcriptional regulation of other katanin subunits by Elk-1 has not been explored, and their analysis is likely to advance our understanding of katanin regulation in various developmental and disease contexts.

Protein posttranslational modifications can function as molecular switches to regulate protein localization, activity, interactions, and abundance (Lee and Yaffe, 2016). In particular, phosphorylation and ubiquitylation are important for regulating katanin localization, microtubule binding, microtubule-severing activity, and levels (Cummings et al., 2009; Loughlin et al., 2011). For example, in humans, phosphorylation of KATNA1 at S42, S109, and T133 by the dual specificity tyrosine-regulated kinase 2 (DYRK2) targets KATNA1 for degradation via the DYRK2-EDD-DDB1/VPRBP (DYRK2-EDVP) E3 ubiquitin ligase complex (Maddika and Chen, 2009). These phosphorylation and ubiquitination events are required for proper cell cycle progression and mitotic transition (Table 2; Maddika and Chen, 2009).

**TABLE 2:** Posttranslational regulation of katanin.

Katanin	Organism	Modified residue(s)	Kinase/phosphatase	Ubiquitin ligase/ligase adapter	Information	References
KATNA1	*H. sapiens*	S131	AURKB*		Regulates KATNA1 activity at kinetochores.	Advani et al., 2018; PMID: 30176123
KATNA1	*H. sapiens*	S42, S109, T133	DYRK2		Regulates KATNA1 levels for cell cycle progression and mitotic function.	Maddika and Chen, 2009; PMID: 19287380
KATNA1	*X. laevis*	S131	AURKB*		Inhibits KATNA1 in a concentration-dependent manner during mitosis by disrupting the ATPase cycle.	Whitehead et al., 2013; PMID: 23178168
KATNA1	*X. laevis*	S131	AURKB*		Regulates KATNA1 activity during scaling of mitotic spindle.	Loughlin et al., 2011; PMID: 22153081
MEI-1	*C. elegans*	S90, S92, S113, S137	MBK-2		MEI-1 S92 phosphorylation promotes its binding to MEL-26, signals degradation by CRL3^MEL–26^ after meiosis; single phosphorylation at S90, S92, S113, and S137 inhibits MEI-1 microtubule-severing.	Joly et al., 2020; PMID: 32412594
MEI-1	*C. elegans*	S92	PP4^PPFR–1^		Dephosphorylation enhances MEI-1 activity during meiosis.	Gomes et al., 2013; PMID: 23918937
MEI-1	*C. elegans*	Unknown	PP4^PPFR–1^		Dephosphorylation stimulates MEI-1 activity during meiosis.	Han et al., 2009; PMID: 19087961
MEI-1	*C. elegans*	S92	MBK-2		Regulates MEI-1 abundance at meiotic exit by increasing affinity for CRL3^MEL–26^.	Stitzel et al., 2006; PMID: 16338136
MEI-2	*C. elegans*	T32, S68 (if MEI-1 present)	MBK-2		Not reported.	Joly et al., 2020; PMID: 32412594
MEI-1	*C. elegans*	Unknown		Cul3	COP9/signalosome is required for the degradation of MEI-1 after meiosis, likely through regulation of Cul3.	Pintard et al., 2003; PMID: 12781129Kurz et al., 2002; PMID: 11847342
MEI-1	*C. elegans*	Unknown		Cul2, RFL-1, Cul3, MEL-26	MEL-26 levels are kept low in meiosis by Cul2 and RFL-1. In meiosis, MEL-26 regulates MEI-1 activity/abundance; MEI-1 regulation is essential for meiotic cell viability. Following meiosis, MEL-26 eliminates MEI-1 activity. Following meiosis, MEL-26 interacts with Cul3 and MEI-1 to control MEI-1 degradation, *in vitro* and *in vivo*.	Johnson et al., 2009; PMID: 19361490Pintard et al., 2003; PMID: 13679921

*Hypothesized kinase to act on katanin but has not been shown experimentally.

In *C. elegans*, Minibrain kinase 2 (MBK-2) phosphorylates the KATNA1 homolog MEI-1 at multiple serines to inhibit its activity (Table 2). More specifically, MBK-2-mediated phosphorylation of MEI-1 at S92 is necessary and sufficient to target MEI-1 for degradation during the oocyte-to-embryo transition (Table 2; Joly et al., 2020). MBK-2 can also phosphorylate the KATNB1 homolog MEI-2, but the significance of this modification has not been determined (Joly et al., 2020). The ubiquitin ligase adaptor MEL-26 functions in parallel to MBK-2 and is required for MEI-1 degradation (Lu and Mains, 2007). Here, MEL-26 interacts with Cul3 and MEI-1 to promote MEI-1 degradation after meiosis and is important in meiotic cell viability (Table 2; Johnson et al., 2009). In mammalian cells, the Cul3 ubiquitin ligase substrate adaptor Ctb9/KLHDC5 similarly targets KATNA1 for ubiquitin-mediated degradation, likely recruited in response to KATNA1 phosphorylation (Cummings et al., 2009). The exact mechanism of Ctb9/KLHDC5 substrate recognition is still unknown; however, this interaction is important for controlling the abundance of KATNA1 during mitosis and for promoting normal mitotic progression (Cummings et al., 2009).

Direct phosphorylation of KATNA1 also plays an important role in regulating microtubule-severing activity at the kinetochores (Loughlin et al., 2011; Whitehead et al., 2013). In *X. laevis*, phospho-inhibition of KATNA1 at the predicted Aurora B kinase consensus site S131 reduces microtubule-severing activity and leads to an increase in spindle length (Table 2); S131 is within a region that contains multiple predicted phosphorylation sites for mitotic kinases like Polo-like kinase 1, Cyclin-dependent kinase 1, and Aurora A and B kinases (Loughlin et al., 2011; Whitehead et al., 2013); therefore, future studies should address which kinases are directly involved in phosphorylating this region. Future detailed analyses of the transcriptional, posttranscriptional, and posttranslational regulation of each katanin subunit in varied developmental and stress-induced conditions will advance our understanding of the dynamic role(s) that they have in specialized contexts and will inform on how their dysregulation contributes to human disease.

In addition to posttranslational modifications of katanin subunits, posttranslational modifications of tubulin, the building blocks of microtubules, are also known to affect katanin activity. For example, preincubation of microtubules with the *X. laevis* Polo-like kinase 1 homolog (Plx1) increased the rate of KATNA1 microtubule severing five-fold *in vitro*, indicating that microtubule phosphorylation was facilitating severing (McNally et al., 2002). Tubulin acetylation also affects microtubule severing. For example, in rat hippocampal neurons and fibroblasts, elevated or reduced levels of acetylated tubulin render microtubules more or less sensitive to katanin-based severing, respectively (Sudo and Baas, 2010). Although tubulin C-terminal tails (CTTs) are key sites of posttranslational modifications (Gadadhar et al., 2017) and these modifications are known to regulate katanin-mediated severing (Bailey et al., 2015; Zehr et al., 2020), KATNA1 was recently found to interact with microtubules lacking the CTT *in vitro* (Belonogov et al., 2019). Here, KATNA1 depolymerized tubulin polymers by removing mass from the ends in a concentration-dependent but ATP-independent manner (although ATP enhances depolymerization) (Belonogov et al., 2019). These findings present a novel concentration-dependent katanin mechanism by which katanin is able to bind to CTT-deficient tubulin, potentially loosening the bonds between α- and β-tubulin, regardless of the presence of ATP or higher-order assembly.

Protein–protein interactions also regulate katanin subcellular localization. In neurons, phosphorylation of the nuclear distribution protein nudE-like 1 (NDEL-1) by CDK5 or CDK2 is necessary for KATNA1 centrosomal localization (Toyo-Oka et al., 2005). Additionally, the nuclear mitotic apparatus (NuMa) and the putative tumor suppressor LAPSER proteins interact with KATNB1 and are thought to direct the localization of KATNB1 to sister chromatids (Jin et al., 2017). LAPSER is also important for the translocation of KATNB1 to the midbody during cell division (Suko and Maru, 2007). *In vitro* and structural studies of the KATNA1 N-terminus, KATNB1 C-terminus, and the abnormal spindle-like microcephaly associated (ASPM) protein showed that they can form a complex (Jiang et al., 2017). Intriguingly, ASPM and katanin stimulated each other’s activity. Here, ASPM is critical for katanin spindle localization, while the ASPM–katanin complex was important for maintaining proper spindle microtubule dynamics (Jiang et al., 2017). Although the protein–protein interaction networks for the five mammalian katanin subunits have been defined through mass proteomic approaches (Cheung et al., 2016), there is still much to learn about how katanin interacting proteins regulate katanin localization and activity, and the importance of these interactions within various disease contexts.

## Conclusion and Future Perspectives

Microtubule polymers and their dynamic rearrangements are paramount to cell architecture, cell motility, cell polarity, cell division, and cell signaling (Muroyama and Lechler, 2017; Goodson and Jonasson, 2018). As such, the growth, maintenance, and function of microtubules require a complex interplay of proteins that interact with and modify the microtubule network. The katanin family of microtubule-severing enzymes has emerged as important factors for regulating microtubule rearrangements. Intriguingly, as evolution has produced more complex organisms, the diversity of katanin subunits in these organisms has expanded. The greater diversity of katanin A-like and B-like subunits in higher organisms raises important questions with regard to their function. For example, are these subunits redundant? Do they have specialized functions? Or do they have both redundant and specialized functions depending on the context or cell type? Recent studies point to the latter, where these subunits share overlapping functions in some cell types and have specific functions in other specialized cell types and contexts, such as during development, during neurogenesis, in mature neurons, and in germ cells (Smith et al., 2012; Hu et al., 2014; Dunleavy et al., 2017; Hatakeyama and Hayashi, 2018; Gao et al., 2019; Lombino et al., 2019). Defining these overlapping and specific functions for each katanin subunit (and their isoforms) is imperative to understanding their function, how they are regulated, and how their dysregulation promotes disease progression.

While transcriptional regulation and posttranslational modifications are known to affect the levels of KATNA1 and its activity, our understanding of how the remaining mammalian subunits (KATNAL1, KATNAL2, KATNB1, and KATNBL1) are regulated is still lacking. Obtaining a systematic profile of degradation and/or activation mechanisms (via phosphorylation, ubiquitination, etc.) for all katanin subunits (in vertebrates and invertebrates) in diverse cell types across phyla and contexts will provide a more comprehensive understanding of how these enzymes are regulated. Phosphorylation, for example, has been postulated to alter the capacity of katanins to oligomerize and to activate microtubule-severing activity (Whitehead et al., 2013). Therefore, resolving the structures of phosphorylated katanin subunits would provide a valuable insight into the role that phosphates, kinases, and phosphatases play in regulating katanin function and whether phosphorylation states can promote observable structural differences in katanins across phyla. Furthermore, the full-length A–B katanin complex has yet to be solved; outside of the canonical MIT:Con80 domain interactions, much remains to be understood regarding the formation of the entire complex. Similarly, full-length structures of the vertebrate B-subunit remain unresolved. The verified interactions between the katanin A- and B-subunits in vertebrates (KATNA1, KATNAL1, and KATNBL1) (Cheung et al., 2016; Willsey et al., 2018) and the propensity for A- and B-katanin heterodimeric formation (Hartman and Vale, 1999; Faltova et al., 2019) raise questions regarding the possibility of mixed hetero-oligomerized katanin complexes and whether mixed hetero-oligomers serve cell- and or context-specific functions. Moreover, there is also a great need for a microtubule-bound katanin structure, as it could inform on its mechanism of action and resolve microtubule-severing models.

In conclusion, the mammalian family of katanins is an intriguing subject for research, as it intersects with cellular pathways including those critical for development that are dysregulated in human diseases. Further research into katanin subunit redundancies and specificities, protein interactions, holoenzyme structure determination, and mechanisms of regulation will further define their roles in human disease and their potential as therapeutic targets.

## Author Contributions

NL and JT conceptualized the project. NL wrote the original draft. NL and JT wrote, reviewed, and edited the manuscript. NL prepared the figures. EM and HN prepared the tables and data for the figures. All authors contributed to the article and approved the submitted version.

## Conflict of Interest

The authors declare that the research was conducted in the absence of any commercial or financial relationships that could be construed as a potential conflict of interest.

## Publisher’s Note

All claims expressed in this article are solely those of the authors and do not necessarily represent those of their affiliated organizations, or those of the publisher, the editors and the reviewers. Any product that may be evaluated in this article, or claim that may be made by its manufacturer, is not guaranteed or endorsed by the publisher.
